# An Efficient and Secure Certificateless Authentication Protocol for Healthcare System on Wireless Medical Sensor Networks

**DOI:** 10.1155/2013/761240

**Published:** 2013-04-21

**Authors:** Rui Guo, Qiaoyan Wen, Zhengping Jin, Hua Zhang

**Affiliations:** State Key Laboratory of Networking and Switching Technology, Beijing University of Posts and Telecommunications, Beijing 100876, China

## Abstract

Sensor networks have opened up new opportunities in healthcare systems, which can transmit patient's condition to health professional's hand-held devices in time. The patient's physiological signals are very sensitive and the networks are extremely vulnerable to many attacks. It must be ensured that patient's privacy is not exposed to unauthorized entities. Therefore, the control of access to healthcare systems has become a crucial challenge. An efficient and secure authentication protocol will thus be needed in wireless medical sensor networks. In this paper, we propose a certificateless authentication scheme without bilinear pairing while providing patient anonymity. Compared with other related protocols, the proposed scheme needs less computation and communication cost and preserves stronger security. Our performance evaluations show that this protocol is more practical for healthcare system in wireless medical sensor networks.

## 1. Introduction

Wireless medical sensor networks (WMSNs) have a capability of connecting patient with doctor by using of lightweight devices with limited memory, small and low power [1]. All these medical sensors collaborate together to collecting patient's physiological signals (e.g., blood pressure, blood sugar, and pulse oximeter) and send the collected data to health professional's hand-held devices (i.e., PDA, iPhone, iPad, etc.) via a wireless channel. The doctor uses these hand-held devices to observe the patient's real-time health condition. 

However, the healthcare system on WMSN has many challenges, such as reliable data transmission, timely delivery of data, and power management [2]. Patient's privacy, a big concern for healthcare system, must be ensured at all sections on WMSN. The Health Insurance Portability and Accountability Act (HIPAA) of 1996 established rules for healthcare provider that it is necessary to control who is accessing to medical server's (MS's) resources and whether they are authorized to do so. Therefore, a secure authentication scheme among patient, MS, and doctor is needed to protect the patient's privacy. So far many schemes that use cryptography have been proposed for this goal. 

 Most recently, Pu et al. [3] proposed a generic construction of smart card-based password authentication protocol for Telecare Medicine Information Systems (TMIS) and proved its security. Wu et al. [4] proposed a concrete efficient authentication scheme for TMIS. In their scheme, Wu et al. introduced a precomputing phase to compute costly and time-consuming exponential operations that are stored in a smart card. He et al. [5] pointed out that Wu et al.'s scheme could not resist impersonation attack and insider attack. Then, they proposed a more secure authentication scheme for TMIS. However, Wei et al. [6] demonstrated that both of Wu et al.'s scheme and He et al.'s scheme could not achieve a two-factor authentication. To overcome the weakness, Wei et al. proposed an improved authentication scheme for TMIS. Zhu [7] showed that Wei et al.'s scheme is vulnerable to an offline password guessing attack and also proposed a new authentication scheme for TMIS.

 A common property of the above schemes is that the patient's identity ID is transmitted in plaintext on the public channel, which leads to impersonating attack and divulging the patient's privacy. To avoid these risks, based on the identity-based public key cryptography (ID-PKC) [8], Das et al. [9] proposed a dynamic ID-based remote client authentication scheme without any verifier table. However, Chien and Chen [10] pointed out that it fails to protect the anonymity of a user, and Ku and Chang [11] demonstrated that it is vulnerable to impersonation attack.

To address the key escrow problem [8] in ID-based authentication scheme, Xiong et al. [12] and Zhang et al. [13] proposed two certificateless authentication schemes, respectively. Unfortunately, their schemes are based on the bilinear pairing. Chen et al. [14] pointed out that the relative computation cost of the bilinear pairing is approximately twenty times higher than that of the scalar multiplication over a cyclic additive group, which is unsuitable for healthcare system on WMSN with lower computation power. Therefore, it is vitally important to present a certificateless authentication without bilinear pairing in the healthcare system.

In this paper, based on certificateless public key cryptography (CL-PKC) [15], we propose a certificateless authentication scheme without bilinear pairing in healthcare system on WMSN. Our protocol can establish a secure channel in Patient-to-MS and Doctor-to-MS with high efficiency. The proposed scheme has the following advantages: (1) it limits the power of MS to resist the malicious MS attack. (2) It ensures that the serial numbers of patient's wearable medical sensor and doctor's hand-held device can be updated in time. (3) It avoids the management of digital certificate and releases the key escrow problem by MS. (4) It achieves the Girault trust level 3 [16] as in traditional public key infrastructure (PKI). (5) It provides patient anonymity. (6) It preserves the perfect forward secrecy. (7) It can resist replay attack and impersonation attack. (8) It does not need to operate the bilinear pairing.

The remainder of this paper is organized as follows.  Section 2 addresses some preliminaries such as the computational assumptions, security model, Girault's trust level, and the model of certificateless authentication.  Section 3 proposes a certificateless authentication scheme and analyzes its security.  Section 4 compares the proposed scheme with some other related schemes. Finally, we conclude the paper in Section 5.

## 2. Preliminaries 

In this section, we review some fundamental backgrounds required in this paper, namely, computational assumptions, security model, Girault's trust level, and the model of certificateless authentication.

### 2.1. Computational Assumptions

The security of our protocol is based on the following computational assumptions:

Discrete Logarithm (DL) problem: let *G* be a cyclic additive group of prime order *p*; *P* is a generator of *G*. Given *Q* ∈ *G*, find an integer *x* ∈ *Z*_p_* such that *Q* = *xP*.

The DL assumption is that there is no polynomial time algorithm that can solve the DL problem with nonnegligible probability.

Computational Diffie-Hellman (CDH) problem: let *G* be a cyclic additive group of prime order *p*; *P* is a generator of *G*. Given *Q*, *R* ∈ *G* and *Q* = *xP*,  *R* = *yP* for any *x*, *y* ∈ *Z*_p_*, compute *x* *yP*.

The CDH assumption is that there is no polynomial time algorithm that can solve CDH problem with nonnegligible probability.

### 2.2. Security Model

In WMSN, we assume that attackers are “internal adversary” and “external adversary.” Internal adversary is a legitimate member of WMSN, such as the malicious MS who has the ability of obtaining the private key and eavesdropping the privacy information of patient. We also assume that the external adversary is divided into four kinds. Type I adversary may capture the transmitted information between patient and doctor. By this information, Type I adversary can get the specific identity of patient. Type II adversary has a capability of extracting the secret key from the transmitted information; it may derivate the secret key in previous session by using this extracted key. Type III adversary may eavesdrop the transmitted information in public channel. Then, it transmits this information again to deceive patient (or doctor) that is provided from the legitimate doctor (or patient). Type IV adversary may capture the transmitted information and extract some important data from it. After that, it may impersonate the patient (or doctor) to communicate with the legitimate doctor (or patient).

### 2.3. Girault's Trust Level

Girault's trust level provides the trust hierarchy for public key cryptography, which can be used to judge the creditability of the authority (e.g., the MS in the healthcare system on WMSN).

 Level 1: the authority knows (or can easily compute) users' secret keys. Therefore, the authority can impersonate any user at any time without being detected.
 Level 2: the authority does not knows (or cannot easily compute) users' secret keys. Nevertheless, it can still impersonate user by generating false guarantees (e.g., false public keys).
 Level 3: the authority cannot compute users' secret keys, and it can be proven that it generates false guarantees of users' if it does so.

According to these definitions, we can easily find that the conventional certificateless cryptography can reach Level 2, and a traditional PKI can achieve Level 3 while the ID-PKC falls into Level 1.

### 2.4. Model of Certificateless Authentication

A certificateless authentication scheme consists of six probabilistic, polynomial time algorithms: *Setup*, *User-Key-Generation*, *Partial-Key-Extract*, *Set-Private-Key*, *Set-Public-Key,* and *Authentication*. These algorithms are defined as follows.

*Setup. *Taking security parameter *k* as input, the authority returns a list of public parameters param and a randomly chosen master secret key msk.

*User-Key-Generation.* Taking a list of public parameters param as input, the user returns a secret key sk and a public key pk.

*Partial-Key-Extract.* Taking param,  msk, user's identity ID, and pk received from the user as inputs, the authority returns a partial private key *D*_ID_ and a partial public key *P*_ID_. 

*Set-Private-Key.* Taking param, *D*_ID_, and sk as inputs, the user returns a private key SK_ID_. 

*Set-Public-Key.* Taking param, *P*_ID_, and pk as inputs, the user returns a public key PK_ID_. 

*Authentication.* Taking identity, private key of the sender, and a list of parameters param as inputs, the receiver verifies the legality of the sender by its public key. 

This model is similar to that of [15] but with a crucial difference that *User-Key-Generation* algorithm must be run prior to the *Partial-Key-Extract* algorithm, which makes the scheme achieve Girault's trust level 3.

## 3. Our Protocol

In this section, we propose a certificateless authentication scheme without bilinear pairing to ensure the legality of Patient and Doctor by the MS.

### 3.1. Construction

The proposed scheme involves three entities: Patient, Doctor, and MS. Before Patient obtains the wearable medical sensor at the first time, MS presets the {ID_*P*_, *S*_*P*_} ∈ {0,1}^*m*^ and {ID_*D*_, *S*_*D*_} ∈ {0,1}^*m*^ into Patient's sensor and his/her doctor's health professional hand-held device through the secure channel as their identities and the serial numbers of equipments, respectively. Besides, these two serial numbers will be preserved secretly by themselves. The details of our certificateless authentication scheme are as follows.

**Figure 1:**
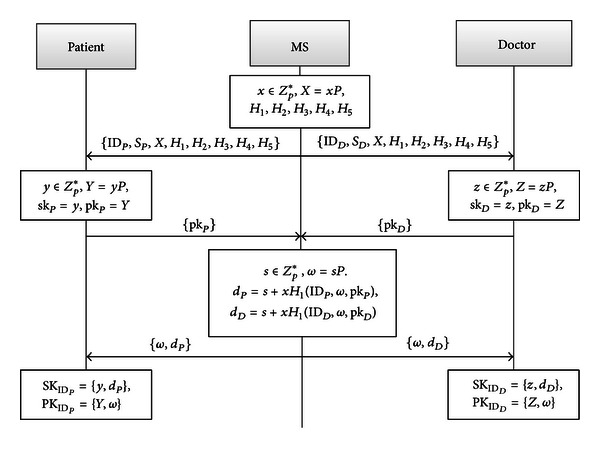
Initialization phase.

We show the initialization phase of this protocol in Figure 1.

*Setup.* The MS generates a large prime *p*, which makes the DL and CDH problems in the cyclic additive group *G* with generator *P* of order *p* be intractable. Then, the MS picks *x* ∈ *Z*_p_* uniformly at random, computes *X* = *xP*, and chooses hash functions

(1) $$\begin{array}{c} H_{1}:{\left\{0,1\right\}}^{m}\times G^{*}\times G^{*}\to Z_{p}^{*},\\ H_{2}:{\left\{0,1\right\}}^{m}\times {\left\{0,1\right\}}^{m}\times {\left\{0,1\right\}}^{m}\to Z_{p}^{*},\\ H_{3}:G^{*}\to {\left\{0,1\right\}}^{m},\;\;H_{4}:{\left\{0,1\right\}}^{m}\to {\left\{0,1\right\}}^{m},\\ H_{5}:{\left\{0,1\right\}}^{m}\to {\left\{0,1\right\}}^{*}, \end{array}$$

which can be achieved easily by collision-resistant hash function. Return {*p*, *P*, *G*, *X*, *H*_1_, *H*_2_, *H*_3_, *H*_4_, *H*_5_} as scheme parameters and the master secret key msk = {*x*}. 

*Patient/Doctor-Key-Generation.* The Patient and the Doctor pick *y*, *z* ∈ *Z*_p_* at random, compute *Y* = *yP*,  *Z* = *zP*, and return (sk_*P*_, pk_*P*_) = (*y*, *Y*) and (sk_*D*_, pk_*D*_) = (*z*, *Z*), respectively. 

*Partial-Key-Extract. *The MS picks *s* ∈ *Z*_p_* at random and computes

(2) $$\begin{array}{c} \omega =sP,\\ d_{P}=s+xH_{1}\left(\text{I}{\text{D}}_{P},\omega ,\text{p}{\text{k}}_{P}\right),\\ d_{D}=s+xH_{1}\left(\text{I}{\text{D}}_{D},\omega ,\text{p}{\text{k}}_{D}\right). \end{array}$$

Return (*P*, *D*_ID_*P*__) = (*ω*, *d*_*P*_), (*P*, *D*_ID_*D*__) = (*ω*, *d*_*D*_) as partial keys to be placed into Patient's sensor and the Doctor's hand-held device, respectively. 

*Set-Private-Key.* The Patient sets SK_ID_*P*__ = (sk_*P*_, *D*_ID_*P*__) = (*y*, *d*_*P*_) as his/her private key, and the Doctor sets SK_ID_*D*__ = (sk_*D*_, *D*_ID_*D*__) = (*z*, *d*_*D*_) as his/her private key as well.

*Set-Public-Key.* Set PK_ID_*P*__ = (pk_*P*_, *ω*) and PK_ID_*D*__ = (pk_*D*_, *ω*) as the public keys of Patient and Doctor, respectively.

**Figure 2:**
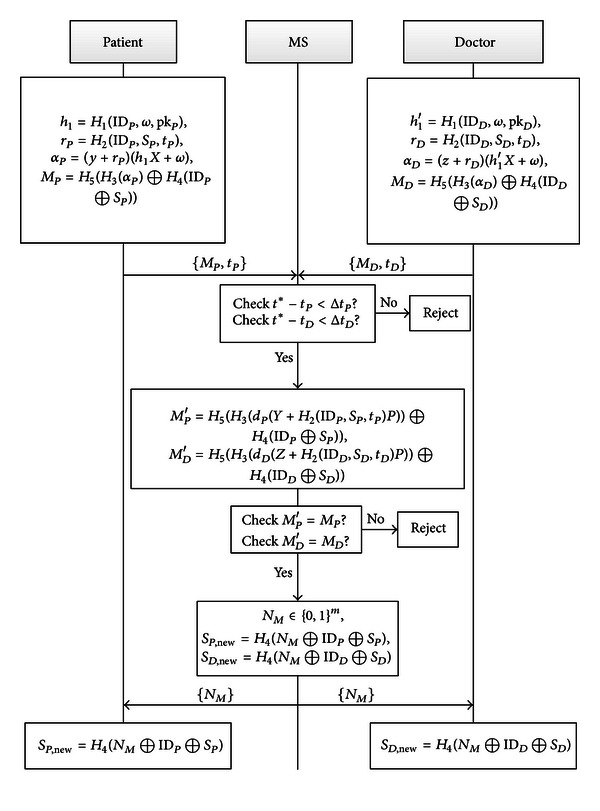
Authentication phase.

Now, we show the authentication phase in Figure 2.

*Authentication*

**Step**

The Patient picks the current time stamp *t*_*P*_ and computes

(3) $$\begin{array}{c} h_{1}=H_{1}\left(\text{I}{\text{D}}_{P},\omega ,\text{p}{\text{k}}_{P}\right),\;\;\;r_{P}=H_{2}\left(\text{I}{\text{D}}_{P},S_{P},t_{P}\right),\\ \alpha_{P}=\left(y+r_{P}\right)\cdot \left(h_{1}X+\omega \right),\\ M_{P}=H_{5}\left(H_{3}\left(\alpha_{P}\right)⊕H_{4}\left(\text{I}{\text{D}}_{P}⊕S_{P}\right)\right). \end{array}$$

Send {*M*_*P*_, *t*_*P*_} to the MS.

**Step 2**

The Doctor picks the current time stamp *t*_*D*_ and computes

(4) $$\begin{array}{c} h_{1}^{\prime }=H_{1}\left(\text{I}{\text{D}}_{D},\omega ,\text{p}{\text{k}}_{D}\right),\;\;r_{D}=H_{2}\left(\text{I}{\text{D}}_{D},S_{D},t_{D}\right),\\ \alpha_{D}=\left(z+r_{D}\right)\cdot \left(h_{1}^{\prime }X+\omega \right),\\ M_{D}=H_{5}\left(H_{3}\left(\alpha_{D}\right)⊕H_{4}\left(\text{I}{\text{D}}_{D}⊕S_{D}\right)\right). \end{array}$$

Send {*M*_*D*_, *t*_*D*_} to the MS.

**Step 3**

If (t* − *t*_*P*_) < Δ*t*_*P*_ and (t* − *t*_*D*_) < Δ*t*_*D*_, where Δ*t*_*P*_ and Δ*t*_*D*_ denote the expected valid time interval for time delay of Patient and Doctor, the MS proceeds to the next step. Otherwise, return “*Reject*.”

**Step 4**

The MS computes

(5) MP′=H5(H3(dP·(Y+H2(IDP,SP,tP)·P))     ⊕H4(IDP⊕SP)),MD′=H5(H3(dD·(Z+H2(IDD,SD,tD)·P))     ⊕H4(IDD⊕SD)).

If *M*_*P*_′ is equal to *M*_*P*_, Patient is a legal one. Otherwise, return “*Reject*.” In addition, if *M*_*D*_′ is equal to *M*_*D*_, Doctor is a legal one. Otherwise, return “*Reject*.”

**Step 5**

The MS picks *N*_*M*_ ∈ {0,1}^*m*^ uniformly at random and updates the serial numbers of Patient and Doctor as follows:

(6) $$\begin{array}{c} S_{P,\text{new}}=H_{4}\left(S_{P}⊕N_{M}⊕\text{I}{\text{D}}_{P}\right),\\ S_{D,\text{new}}=H_{4}\left(S_{D}⊕N_{M}⊕\text{I}{\text{D}}_{D}\right). \end{array}$$

Send {*N*_*M*_} to Patient and Doctor.

**Step 6**

By using of {*N*_*M*_}, Patient computes

(7) $$\begin{array}{c} S_{P,\;\text{new}}=H_{4}\left(S_{P}⊕N_{M}⊕\text{I}{\text{D}}_{P}\right) \end{array}$$

for updating the serial number of his/her wearable medical sensor.

**Step 7**

After obtaining {*N*_*M*_}, Doctor computes

(8) $$\begin{array}{c} S_{D,\text{ new}}=H_{4}\left(S_{D}⊕N_{M}⊕\text{I}{\text{D}}_{D}\right) \end{array}$$

for updating the serial number of his/her hand-held device.

### 3.2. Security Analysis

**Theorem 1**

This certificateless authentication scheme is secure in the following possible attacks, provided that *H*_1_ is a collision-resistance hash function and DL and CDH problems are intractable.

*Proof*

*Anonymity.* In the proposed scheme, the partial key *d*_*P*_ = *s* + *xH*_1_(ID_*P*_, *ω*, pk_*P*_) is used instead of ID_*P*_ to ensure the Patient's anonymity. Since ID_*P*_ is never transmitted as plaintext form in the public channel, Type I adversary cannot find the real identity ID_*P*_ of Patient. That is, when Patient transmits his/her health information, their real identity ID_*P*_ can only be computed as *d*_*P*_ = *s* + *xH*_1_(ID_*P*_, *ω*, pk_*P*_) to be transmitted, where *s* is a random value, *H*_1_ is a collision-resistant hash function, and *x* is the master secret key which is preserved by MS. Therefore, Type I adversary cannot trace Patient. 

*Perfect Forward Secrecy.* To extract {*M*_*P*_, *M*_*D*_} without the knowledge of the values {*r*_*P*_, *y*, *d*_*P*_, *r*_*D*_, *z*, *d*_*D*_}, Type II adversary should solve the DL problem and the CDH problem from public parameters. Moreover, *r*_*P*_ = *H*_2_(ID_*P*_, *S*_*P*_, *t*_*P*_) and *r*_*D*_ = *H*_2_(ID_*D*_, *S*_*D*_, *t*_*D*_) will be different in every session for the reason of time stamps {*t*_*P*_, *t*_*D*_} and the updated serial numbers {*S*_*P*_, *S*_*D*_}. Therefore, Type II adversary cannot receive the previous value {*r*_*P*_, *y*, *d*_*P*_, *r*_*D*_, *z*, *d*_*D*_} and the protocol enjoys the perfect forward security.

*Replay Attack.* During the data transmission, Type III adversary may eavesdrop {*M*_*P*_,  *M*_*D*_} and impersonate the legitimate Patient and Doctor to transmit {*M*_*P*_,  *M*_*D*_} to MS. After each session is over, the serial numbers of the Patient's sensor and Doctor's hand-held device have been updated to be the new serial numbers {*S*_*P*, new_,  *S*_*D*, new_}, which can be used to generate the new messages  {*M*_*P*, new_,  *M*_*D*, new_}. Hence, Type III adversary cannot pass the verification by retransmitting {*M*_*P*_, *M*_*D*_} in the new session. Moreover, there are time stamps {*t*_*P*_, *t*_*D*_} in this scheme, which ensures the freshness of {*M*_*P*_, *M*_*D*_}. 

*Impersonation Attack.* The impersonation attack fails due to the secret serial number. Provided that Type IV adversary wants to impersonate the legitimate Patient and Doctor, it must produce the relative {*M*_*P*_, *M*_*D*_} for passing the verification of MS. However, in order to generate the exactly {*M*_*P*_, *M*_*D*_}, Type IV adversary needs to obtain the current serial numbers {*S*_*P*_, *S*_*D*_} first of all, which are preserved secretly by Patient and Doctor and updated in time in the end of *Authentication* phase. Therefore, Type IV adversary has no capability to impersonate the legitimate Patient and Doctor to generate the correct {*M*_*P*_, *M*_*D*_}.

*Malicious MS Attack. *The malicious MS cannot obtain the private keys to eavesdrop the privacy information of patient. This authentication scheme is proposed on the base of CL-PKC, and the private keys (SK_ID_*P*__, SK_ID_*D*__) generated by Patient and Doctor consist of partial private keys (*d*_*P*_, *d*_*D*_) and the secret values (*y*, *z*). The malicious MS cannot obtain (*y*, *z*) from public parameters for the intractable of DL and CDH problems. Therefore, our scheme can resist the malicious MS attack.

*Achieve Girault's Trust Level 3. *The *Patient/Doctor-Key-Generation* must be run prior to *Partial-Key-Extract*. In this way, the *Partial-Key-Extract* algorithm includes (pk_*P*_, pk_*D*_) generated by Patient and Doctor as input. Therefore, provided that the MS replaces (pk_*P*_, pk_*D*_), there will exist two working keys (pk_*P*_, pk_*P*_′) and (pk_*D*_, pk_*D*_′) for Patient and Doctor, respectively. Furthermore, two working public keys (PK_ID_*P*__, PK_ID_*P*__′) binding only one identity ID_*P*_ can result from two partial private keys (the same to Doctor), and only the MS could generate these two working partial private keys. Hence, it can be proven that MS generates false guarantees of Patient and Doctor, which means that our scheme achieves Girault's trust level 3 (the same level as is enjoyed in a traditional PKI).

Thus, to sum up the analysis above, we complete the proof of Theorem 1.

## 4. Comparisons

In this section, we evaluate some performance issues of our protocol with related works in functionality and efficiency.

### 4.1. Functionality Comparisons

Table 1 demonstrates the functionality comparisons between the proposed scheme and others [7, 12, 13]. Zhu's, Xiong et al.'s, and Zhang et al.'s protocols do not provide user anonymity. Moreover, the schemes in [12, 13] are insecure against the replay attack. However, as shown in Table 1, our scheme not only provides user anonymity but also achieves all security requirements. Furthermore, our scheme does not need an additional certificate to bind the user to its public key.

### 4.2. Efficiency Comparisons

In this subsection, we compare the proposed scheme with others on the computation complexity of authentication (Authen), bandwidth of the largest message (Bandwidth), and operation time in authentication (Time). Without considering the addition of two points, hash function and exclusive-OR operations, each scheme has three types of operations, that is, pairing (P), exponentiation (E), and scalar multiplication (S).

We evaluate the cryptographic operations by using of MIRACL (version 5.6.1, [17]), a standard cryptographic library, on a laptop using the Intel Core i5-2400 at a frequency of 3.10 GHz with 3 GB memory, and then obtain the average running time in Table 2. For pairing-based schemes, we use the Fast-Tate-Pairing in MIRACL, which is defined over the MNT curve *E*/*F*_*q*_ [18] with embedding degree 4, and *q* is a 160-bit prime. For ECC-based scheme, we employed the parameter secp192r1 [19], where *p* = 2^192^ − 2^64^ − 1. Moreover, the length of an element in multiplication group is set to be 1024 bits.

We compare the computation cost of different protocols with the method in [20]. For example, to finish the authentication in [12], six pairing operations, six exponentiations in *Z*_p_*, and twenty-one scalar multiplications are needed; thus, the operation time is 2.66 × 6 + 3.75 × 6 + 0.94 × 21 = 58.2 ms. Assuming the bit size of the identity, the point in additional group and the output of one-way hash function are all 192 bits. We also assume that the size of timestamp is 32 bits. In [12], the largest message contains three points in additional group and one identification; thus, the bandwidth of it is (192 × 3 + 192)/8 = 96 bytes. The detailed comparison results are demonstrated in Table 3. 

From Table 3, we know that the largest bandwidth of our scheme is only 28 bytes and the whole operation time in authentication is only 7.52 ms, which shows that our protocol is suitable for the lightweight devices (with limited memory, small and low power) in the healthcare system on WMSN.

## 5. Conclusions

In this paper, we propose a secure certificateless authentication scheme to ensure the legality of Patient and Doctor in healthcare system on WMSN. Meanwhile, this protocol also provides patient anonymity and resists the malicious MS attack to meet the privacy requirements in HIPAA. Our certificateless authentication protocol achieves a lower communication and computational overhead and stronger security than others. By the performance evaluation, the results show that our protocol is suitable for healthcare system on WMSN.

## Figures and Tables

**Table 1:** Functionality comparisons.

Properties	[7]	[12]	[13]	Ours
User anonymity	No	No	No	Yes
Perfect forward secrecy	No	Yes	Yes	Yes
Replay attack resistance	Yes	No	No	Yes
Impersonation attack resistance	Yes	Yes	Yes	Yes
Malicious server attack resistance	Yes	Yes	Yes	Yes
No certificate management	No	Yes	Yes	Yes
Trust level	1	2	3	3

**Table 2:** Cryptographic operation time.

Fast-Tate-Pairing	Exponential	Scalar multiplication
2.66 ms	3.75 ms	0.94 ms

**Table 3:** Efficiency comparisons.

Scheme	Authen	Bandwidth	Time
[7]	4E	48 bytes	15 ms
[12]	6P + 6E + 21S	96 bytes	58.2 ms
[13]	2P + 10S	72 bytes	14.72 ms
Ours	8S	28 bytes	7.52 ms
